# Differential MicroRNA Expression Profile in Myxomatous Mitral Valve Prolapse and Fibroelastic Deficiency Valves

**DOI:** 10.3390/ijms17050753

**Published:** 2016-05-18

**Authors:** Yei-Tsung Chen, Juan Wang, Abby S. Y. Wee, Quek-Wei Yong, Edgar Lik-Wui Tay, Chin Cheng Woo, Vitaly Sorokin, Arthur Mark Richards, Lieng-Hsi Ling

**Affiliations:** 1 Cardiovascular Research Institute, National University Health System, Singapore 119228, Singapore; mdcwaju@nus.edu.sg (J.W.); abbiwsy@hotmail.com (A.S.Y.W.); arthur_mark_richards@nuhs.edu.sg (A.M.R.); lieng_hsi_ling@nuhs.edu.sg (L.-H.L.); 2 Department of Medicine, Yong Loo Lin School of Medicine, National University of Singapore, Singapore 117597, Singapore; 3 Department of Cardiology, Tan Tock Seng Hospital, Singapore 308433, Singapore; quek_wei_yong@ttsh.com.sg; 4 Department of Cardiology, National University Heart Centre, Singapore 119228, Singapore; edgar_tay@nuhs.edu.sg; 5 Department of Surgery, Yong Loo Lin School of Medicine, National University of Singapore, Singapore 117597, Singapore; surwoocc@nus.edu.sg (C.C.W.); vitaly_sorokin@nuhs.edu.sg (V.S.); 6 Christchurch Heart Institute, University of Otago, Christchurch 8014, New Zealand

**Keywords:** degererative mitral valve disease (DMVD), myxomatous mitral valve prolapse (MMVP), fibroelastic deficiency (FED), microRNA

## Abstract

Myxomatous mitral valve prolapse (MMVP) and fibroelastic deficiency (FED) are two common variants of degenerative mitral valve disease (DMVD), which is a leading cause of mitral regurgitation worldwide. While pathohistological studies have revealed differences in extracellular matrix content in MMVP and FED, the molecular mechanisms underlying these two disease entities remain to be elucidated. By using surgically removed valvular specimens from MMVP and FED patients that were categorized on the basis of echocardiographic, clinical and operative findings, a cluster of microRNAs that expressed differentially were identified. The expressions of has-miR-500, -3174, -17, -1193, -646, -1273e, -4298, -203, -505, and -939 showed significant differences between MMVP and FED after applying Bonferroni correction (*p* < 0.002174). The possible involvement of microRNAs in the pathogenesis of DMVD were further suggested by the presences of *in silico* predicted target sites on a number of genes reported to be involved in extracellular matrix homeostasis and marker genes for cellular composition of mitral valves, including decorin (DCN), aggrecan (ACAN), fibromodulin (FMOD), α actin 2 (ACTA2), extracellular matrix protein 2 (ECM2), desmin (DES), endothelial cell specific molecule 1 (ESM1), and platelet/ endothelial cell adhesion molecule 1 (PECAM1), as well as inverse correlations of selected microRNA and mRNA expression in MMVP and FED groups. Our results provide evidence that distinct molecular mechanisms underlie MMVP and FED. Moreover, the microRNAs identified may be targets for the future development of diagnostic biomarkers and therapeutics.

## 1. Introduction

Valvular heart disease (VHD) is an important public health concern with an age-dependent prevalence [1,2]. In developed countries, degenerative mitral valve disease (DMVD) is the most common cause of mitral regurgitation (MR) and, by far, the leading indication for mitral valve surgery [2]. DMVD can be classified into two subtypes on the basis of clinical patterns, imaging findings and gross surgical appearances: (1) billowing mitral valve (alternatively known as Barlow’s disease or myxomatous mitral valve prolapse (MMVP)), characterized by excessive, diffuse accumulation of glycosaminoglycan material; and (2) fibroelastic deficiency (FED) which is believed to be caused by disturbances in the homeostasis of connective tissue production [3,4,5,6,7].

Currently, echocardiography is the principal diagnostic modality used to discriminate MMVP and FED. MMVP patients tend to be younger, have diffusely thickened, redundant leaflets, multisegmental prolapse, and mitral annular dilatation and often disjunction. By contrast, the FED patient is often older, has more focal disease, typically a flail segment with a ruptured chord, and no or mild annular dilatation [4,5,8]. Histopathological differences have also been reported. MMVP valves often manifest a disproportionately thickened spongiosa layer infiltrated with mucopolyaccharides and altered collagen composition, while FED valves are relatively thinner, with classical four-layer valvular architecture and predominant elastic fiber alterations [6,9]. However, there is considerable overlap [9], and, in one study of 130 mitral valve specimens, these entities could not be reliably distinguished by qualitative histopathology alone [6]. Furthermore, in FED, changes of fibroelastic deficiency are often interspersed with focal areas of primary or even secondary myxomatous change [10].

Genetic influences may also differ, being more established in MMVP compared to FED. Early genetic linkage studies mapped MMVP loci to chromosomes 16p11.2-p12.2 (MMVP1), 11p15.4 (MMVP2), 13q31.3-q32.1 (MMVP3), and Xq28 [11,12,13,14]. More recently, genetic analyses of a large multigenerational family led to the discovery of familial missense and deleterious mutations in the Drosophila cell polarity gene (DCHS 1) and subsequently attenuated its protein stability and led to abnormal mitral valve morphogenesis [15]. A meta-analysis of two genome-wide association studies with large nonsyndromic MVP and nondiseased control cohorts reported the discovery of six novel MMVP loci [16]. Ablation of the candidate genes, LIM and cysteine-rich domains 1 (LMCD1) and tensin 1 (TNS1), in genetically engineered animal models, led to atrioventricular valve regurgitation and abnormal mitral valve morphogenesis [16].

Because the biological mechanisms underlying MMVP and FED remain poorly understood, surgical intervention is recommended on the basis of severe functional abnormality [17]. The use of echocardiography to further distinguish MMVP from FED is not only of academic interest but clinically important, since repair of MMVP requires greater surgical expertise [5,9,18,19]. However, differentiation of MMVP and FED by echocardiography can be challenging owing to technical limitations, and the existence of “forme frustes” with overlapping features [5]. To improve the accuracy of preoperative diagnosis, it is needful to identify more fundamental and unique pathobiological characteristics of MMVP and FED. MicroRNAs comprise a group of small noncoding RNAs (21–25 nucleotides) that have emerged as the key regulators for gene expression. MicroRNAs are crucial for regulating almost all aspects of biological pathways, including differentiation, proliferation, metabolism, and cell death [20]. Disturbances in microRNA expression have been linked with complex diseases including cardiovascular diseases, as shown by studies from our group [21,22] and others [23,24,25,26]. The role of microRNAs in VHD, however, has only recently been investigated. Using aortic valve tissue and interstitial cells, one research group demonstrated the role of miR-141 in regulating valvular calcification [27]. For DMVD, the roles of microRNAs in valvular development and disease progression remain to be addressed. We hypothesize that microRNA profiles are distinctly different in MMVP and FED. In this study, echocardiography was used to determine DMVD type (MMVP or FED), and human mitral valve tissues obtained during corrective surgery were subjected for gene expression analysis. Using qPCR methods, web-based microRNA target and pathway predictive algorithms, the expressions of a cluster of microRNAs and predicted target genes that were reported to be dysregulated in diseased mitral valves and contributed to the progression of myxomatous accumulation were compared between MMVP and FED cohorts.

## 2. Results

### 2.1. Differential MicroRNA Expression in Myxomatous Mitral Valve Prolapse (MMVP) and Fibroelastic Deficiency (FED) Mitral Valves

The characteristics of the study patients, 10 each with MMVP and FED, are summarized in Table 1 and Table S1. Almost all subjects had severe MR, with similar degrees of left ventricular and left atrial remodeling in both groups (Table 1). MMVP patients had larger mitral annular dimensions than those with FED (*p* = 0.016), even when indexed for body surface area (*p* = 0.019). When compared with FED, MMVP patients had significantly longer and thicker anterior mitral leaflets. Detailed analyses of leaflet morphology showed typical multisegmental involvement in MMVP patients, while most FED patients had an isolated flail segment and chordal rupture (Table S1).

**Figure 1:**
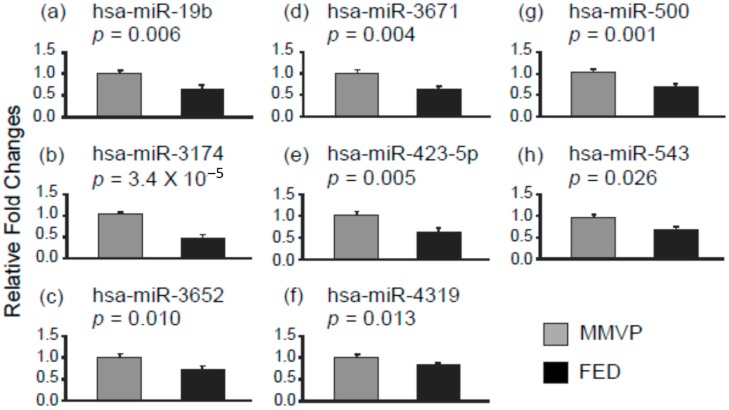
Differential microRNA expression patterns in myxomatous mitral valve prolapse (MMVP) and fibroelastic deficiency (FED) valvular tissues. The expressions of selected microRNAs were examined by using semi-quantitative PCR. (**a**) hsa-miR-19b; (**b**) hsa-miR-3174; (**c**) hsa-miR-3652; (**d**) hsa-miR-3671; (**e**) hsa-miR-423-5p; (**f**) hsa-miR-4319; (**g**) hsa-miR-500; and (**h**) hsa-miR-543. Data are presented as mean ± SEM, Student’s *t*-test, *p* values as indicated, *n* = 10.

**Figure 2:**
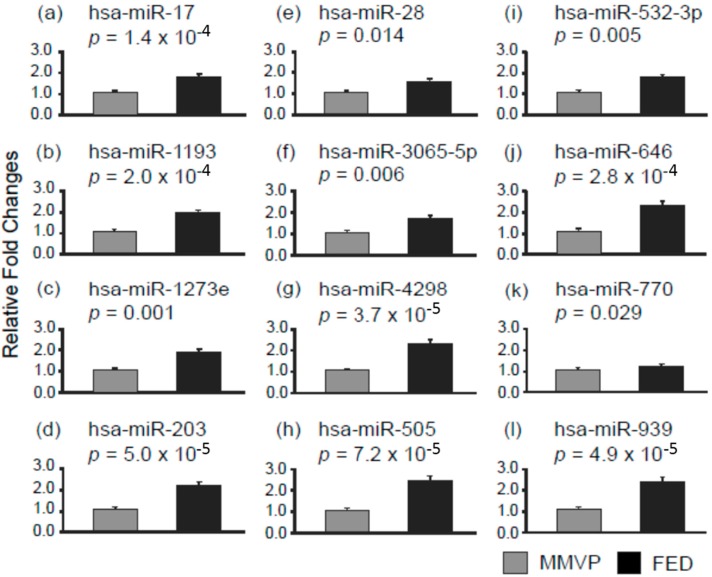
Differential microRNAs expression patterns in MMVP and FED samples. The expressions of selected microRNAs were examined by using semi-quantitative PCR. (**a**) hsa-miR-17; (**b**) hsa-miR-1193; (**c**) hsa-miR-1293e; (**d**) hsa-miR-203; (**e**) hsa-miR-28; (**f**) hsa-miR-3065-5p; (**g**) hsa-miR-4298; (**h**) hsa-miR-505; (**i**) hsa-miR-532-3p; (**j**) hsa-miR-646; (**k**) hsa-miR-770; and (**l**) hsa-miR-939. Data are presented as mean ± SEM, Student’s *t*-test, *p* values as indicated, *n* = 10.

MicroRNA profiling on MMVP and FED specimens (*n* = 3) was first conducted used human whole genome miRNA qPCR profiling kits from Applied Biological Materials (data not shown) to shortlist microRNAs for single microRNA qPCR examination. In this study, 23 microRNA entities differentially expressed in the MMVP and FED profiling study were selected, and the expressions of each microRNAs was examined in MMVP (*n* = 10) and FED (*n* = 10) specimens. Among 23 microRNAs examined, the expressions of 20 microRNAs differed significantly between MMVP and FED cohorts. The expressions of eight microRNAs, namely, hsa-miR-19b, hsa-miR-3174, hsa-miR-3652, hsa-miR-3671, hsa-miR-423-5p, hsa-miR-4319, hsa-miR-500, and hsa-miR-543 were found to be higher in MMVP specimens when compared to FED samples (Figure 1). The expressions of 12 microRNAs, namely, hsa-miR-17, hsa-miR-1193, hsa-miR-1273e, hsa-miR-203, hsa-miR-28, hsa-miR-3065-5p, hsa-miR-4298, hsa-miR-505, hsa-miR-532, hsa-miR-646, hsa-miR-770, and hsa-miR-939 were found to be lower in MMVP specimens when compared to FED samples (Figure 2). Readjusted critical value (Bonferroni correction) for individual test is 0.002174 (0.05/23).

By using DNA intelligent Analysis (DIANA) miRPath v.2.0, a web-based microRNA-targeted pathway analysis algorithm [28], the Kyoto Encyclopedia of Genes and Genomes (KEGG) pathways that might be altered by the 20 microRNAs were identified (Figure S1, Table S2). Hierarchical clustering analysis of targeted pathways and microRNAs further illustrates the relationship of individual microRNAs and their targeted pathways. In this study, 33 KEGG pathways were identified by *a posteriori* statistical analysis (Union of Pathways) embedded in the miRPath v.2.0 program. Pathways that related to glycoprotein synthesis, ubiquitin mediated proteolysis, cell–cell junctions, and cytoskeleton homeostasis, as well as MAPK, Wnt, PI3K-AKT, and ErbB signaling pathways vital for normal cell function, were predicated to be affected by the microRNAs identified (Table S2).

### 2.2. Differential mRNA Expression in MMVP and FED Mitral Valves

To further explore the biological relevance of the microRNAs identified with valvular pathogenesis, genes that have been indicated to correlate with myxomatous development were selected to test the possible 3′UTR/microRNA interaction used computer algorithm. Interestingly, using miRWalk, a web-based *in silico* microRNA-mRNA prediction database [29], a high percentage of genes that have been implicated in MMVP were found to be targeted by microRNAs that found to be differentially expressed in MMVP and FED valves. It is known that microRNAs negatively regulate the expression of their target genes post-transcriptionally. To test whether the expressions of these MMVP related genes were negatively correlated with the expressions of microRNAs observed in this study, qPCR was performed.

**Figure 3:**
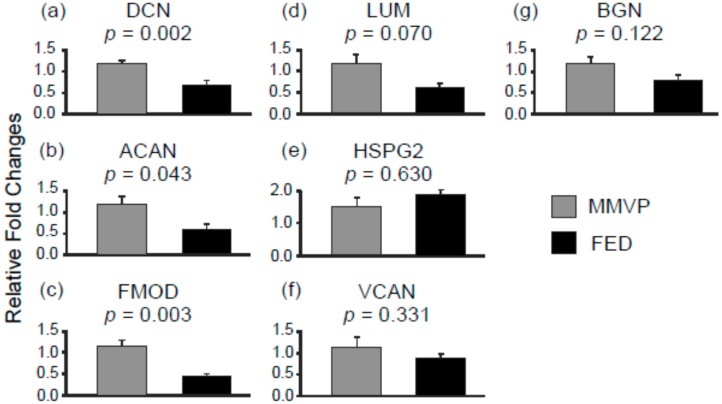
The mRNA expression of proteoglycan genes in MMVP and FED samples. (**a**) DCN, decorin; (**b**) ACAN, aggrecan; (**c**) FMOD, fibromodulin; (**d**) LUM, lumican; (**e**) HSPG2, heparin sulfate proteoglycan 2; (**f**) VCAN, versican; and (**g**) BGN, biglycan. Data are presented as mean ± SEM, Student’s *t*-test, *p* values as indicated, *n* = 10.

The expression of mRNAs for proteoglycans, decorin (DCN), aggrecan (ACAN), and fibromodulin (FMOD), were found to be lower in FED samples when compared with MMVP samples (Figure 3a–c); while no significant differences were observed in the mRNA levels of lumican (LUM), heparin sulfate proteoglycan 2 (HSPG2), versican (VCAN) and biglycan (BGN) between MMVP and FED mitral valve samples (Figure 3d–g). Further comparisons revealed inverse expression patterns between microRNAs and their putative target genes, suggesting that the expression of particular proteoglycans (DCN, ACAN, and FMOD) could be influenced by the differentially expressed microRNA in MMVP and FED valves (Table 2, Figure S2).

**Figure 4:**
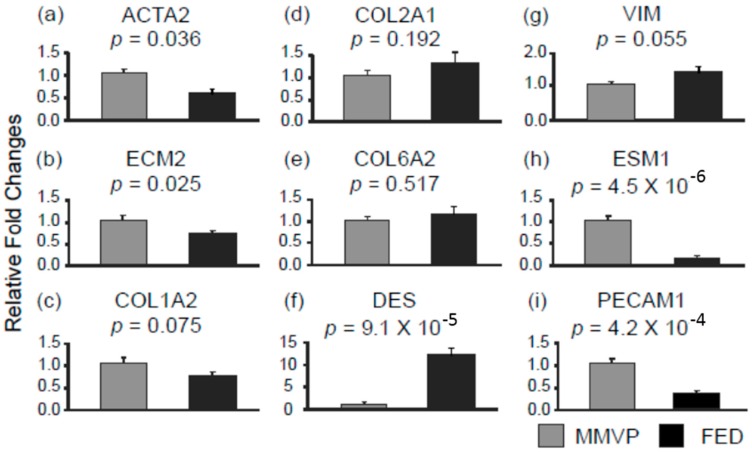
The expressions of structural integrity related genes in MMVP and FED samples. (**a**) ACTA2, α actin 2; (**b**) ECM2, extracellular matrix protein 2; (**c**) COL1A2, collagen type 1 α 2; (**d**) COL2A1, collagen type 2 α 1; (**e**) COL6A2, collagen type 6 α 2; (**f**) DES, desmin; (**g**) VIM, vimentin; (**h**) ESM1, endothelial cell specific molecule 1; and (**i**) PECAM1, platelet/endothelial cell adhesion molecule 1. Data are presented as mean ± SEM, Student’s *t*-test, *p* values as indicated, *n* = 10.

Next, examination of the expression of genes that have been reported to be important for structural integrity revealed lower expressions of α actin 2 (ACTA2) and extracellular matrix protein 2 (ECM2) in FED samples when compared with MMVP samples (Figure 4a,b), whereas, no significant differences were observed in the expressions of different subtype of colleagen genes, collagen type 1 α 2 (COL1A2), collagen type 2 α 1 (COL2A1) and collagen type 6 α 2 (COL6A2) in MMVP and FED samples (Figure 4c–e). Inverse expression patterns observed between microRNA (miR-17, miR-28-5p, and miR-203) and their putative target genes (ECM2, and ACTA2) are consistent with a microRNA-based regulatory effect (Table 3, Figure S2). Additionally, it is known that valvular interstitial cells (VICs) play important roles in the homeostasis of extracellular matrix composition; altered VIC status has been associated with myxomatous deposition in DMVD [30]. Analyses of the expressions of cell lineage marker genes for myofibroblast-like cells, desmin (DES) and vimentin (VIM), showed less expression of these genes in MMVP samples when compared with FED samples (Figure 4f,g). In contrast, the expression of endothelial cell specific molecule 1 (ESM1), and platelet/ endothelial cell adhesion molecule 1 (PECAM1) were found to be higher in MMVP samples when compared with FED samples (Figure 4h,i). Similarly, inverse expression patterns between microRNA and their target genes were observed, suggesting the expressions of cellular markers could also be influenced by the differentially expressed microRNA in MMVP and FED valves (Table 3, Figure S2).

## 3. Discussion

DMVD is a common disease entity in the general population that can potentially lead to severe cardiovascular complications with a high health and economic burden. A growing body of evidence from clinical imaging and pathological studies of DMVD suggest that its two major variants, MMVP and FED, may have distinct biological underpinnings [4,5,6]. The need to properly differentiate MMVP from FED is clinically important as more complex surgical procedures are often required to repair the diffuse myxomatous lesions of MMVP [18]. Elucidating the mechanisms underlying MMVP and FED may not only enrich knowledge of disease pathophysiology, it could contribute to the identification of diagnostic biomarkers and development of therapeutics targeting specific pathogenic signaling pathways. Results from the current study not only support the hypothesis that distinct molecular mechanisms underlie MMVP and FED, but also provide a foundation for deciphering disease causing mechanisms in DMVD, with the focus on microRNAs and corresponding target genes.

The roles of microRNAs in the pathogenesis of cardiovascular diseases and their diagnostic and therapeutic potential have been explored extensively in the past decade. Although DMVD is highly prevalent, to date, the roles of microRNAs in valvular biology remain largely unknown. In this study, we compare, for the first time, the expression of a subset of microRNAs in mitral valve tissue from well-characterized MMVP and FED patients. Results of 20 differentially expressed microRNAs provide evidence suggesting that distinct signaling pathways might underlie MMVP and FED. Among the different microRNA entities, hsa-miR-423-5p is of interest since several human studies have found increased expression in various cardiovascular diseases including heart failure, dilated cardiomyopathy and myocardial infarction [31,32,33]. Since hsa-miR-423-5 has been identified as a circulating biomarker for heart failure, the differential expression of hsa-miR-423-5p in MMVP and FED may reflect different degrees of cardiac remodeling and dysfunction. The other cardiovascular related microRNA, hsa-miR-19b, was reported to be a putative therapeutic target for congenital heart diseases [34], and may be involved in regulating the Wnt/β-catenin signaling pathway, which is an essential regulator of cardiovascular differentiation, morphogenesis and progenitor self-renewal with a key role in cardiac valve formation [35,36]. To our knowledge, no microRNA has been reported to regulate ECM homeostasis in MMVP and/or FED. Our results may in part provide a molecular basis for the distinct valvular architecture observed in MMVP and FED [4,6]. In addition to ECM homeostasis, results from the DIANA miRPath v.2.0 algorithm also suggested the involvement of microRNAs identified in various signaling pathways, including MAPK, Wnt, PI3K-Akt, ErbB, and p53 signaling known to be essential for maintaining proper cellular functions. In this study, a subset of genes that concern the integrity of valvular connective tissues, myxoid deposition, and ECM homeostasis [4,6], as well as valvular cellular composition [30,37,38] were selected to examine possible 3′UTR/microRNAs interaction by using web-based microRNA target predictive algorithms. Interestingly, we found that the expressions of valvular structural integrity genes (DCN, ACAN, FMOD, ACTA2, and ECM2), and valvular cellular marker genes (DES, ESM1, and VIM) were inversely correlated with the expression of microRNAs that pose predicted regulatory effects on them, suggesting a possible microRNA based regulatory mechanism. In future, it will be relevant to further examine the expression of microRNAs and their physiological functions within VIC, as well as in other mitral valve cells, such as valvular endothelial cells.

Collectively, our study reveals not only a cluster of microRNAs that are differentially expressed in MMVP and FED, but also inverse correlations of the expressions of microRNAs with putative target genes, suggesting the involvement of microRNAs in modulating the expression of key genes that are crucial for valvular ECM homeostasis. Future studies could be directed towards further characterization of the biological function of individual microRNAs and examination of how different microRNAs are orchestrated in DMVD.

## 4. Materials and Methods

### 4.1. Mitral Valve Tissues

Mitral valve tissue from patients with MMVP and FED was obtained from the Cardiovascular Tissue Bank/Tissue Repository at the National University Hospital, Singapore. Cases were classified as MMVP or FED based on medical records, operative reports and echocardiographic data, using the criteria outlined above. All clinical data were reviewed independently by three senior cardiologists with expertise in VHD. Of 22 cases adjudicated, there was complete concordance among all three observers on phenotype in 10 MMVP and 10 FED patients, and partial concordance in 2 patients that were excluded from further study. The study protocol was approved by the National Health Group Domain Specific Review Board (Reference code C/11/021). Surgically excised mitral valve tissues were rinsed immediately with cold phosphate buffered saline (PBS) and immersed into RNAlater (Invitrogen, Life Technologies Inc., Frederick, MA, USA) containing tubes for low temperature transportation. Tissues preserved in RNAlater tubes were stored at −80 °C for later extraction of nucleic acid.

### 4.2. Total RNA Extraction and Gene Expression Analysis

Mitral valvular tissues were lysed by gentleMACS™ dissociator (Miltenyi Biotec, Bergisch Gladbach, Germany) in TRIZOL reagent (Invitrogen). Total RNA was extracted according to manufacturer’s protocol, followed by treatment with DNase I (Invitrogen). The RNA quantity and quality were determined using the ND-1000 Spectrophotometer (Nanodrop™, Rockland, DE, USA). Twenty-three microRNA entities used in this study were selected based on pilot microRNA expression profile data obtained by using the whole human genome miRNA qPCR profiling kit from Applied Biological Materials (Richmond, BC, Canada) (ABM) (MA003, Sanger miRBase Version 16). Total RNAs from 3 MMVP samples and 3 FED samples were used for analysis. The data were filtered and analyzed used Microsoft Excel. The differences for individual microRNAs between MMVP and FED were compared by using Student’s *t*-test and sorted according to *p* values. Twenty-three microRNAs with low *p* values (*p* < 0.1) and high fold changes were selected for further Taqman qPCR analysis in current study. Reverse transcription PCR (RT-PCR) and real-time quantitative stem-loop PCR (RT-qPCR). The Taqman High Cap cDNA Rev Trans Kit (Applied Biosystems, Foster City, CA, USA), the Taqman miRNA RT kit (Applied Biosystems), and the Taqman Universal MMIX II with UNG (Applied Biosystems) were utilized to quantify the level of genes or miRNAs of interest. Expression levels for mRNAs and miRNAs were calculated based on their *C*_t_ values using housekeeping genes, β actin, Glyceraldehyde-3-Phosphate Dehydrogenase (GAPDH), glucuronidase β (GUSB) and U6, respectively. RT-qPCR results were processed and analyzed using the comparative CT method (2^−ΔΔ*C*t^) method. The detailed procedure was described previously [22].

### 4.3. Statistical Analysis

Continuous clinical and echocardiographic variables were compared using the independent samples *t*-test and dichotomous variables using the Fischer or chi-square test, whichever was appropriate. Data for clinical characteristic of study cohorts were expressed as mean ± standard error of mean (SEM) or number (percentage). Relative expression of genes of interest between MMVP and FED patients were compared using Student’s *t*-test (type 3). These data were expressed as mean ± SEM. The significance level of mRNA comparison was set at *p* < 0.05. Bonferroni correction was applied to control the familywise error rate of the comparison of microRNA expressions. The adjusted critical value is (0.05/23) 0.002174.

## Figures and Tables

**Table 1:** Clinical and echocardiographic findings in patients with myxomatous mitral valve prolase (MMVP) and fibroelastic deficiency (FED).

Clinical Information	MMVP (*n* = 10)	FED (*n* = 10)	*p* Value
Age, years	57.50 ± 3.76	59.10 ± 3.05	0.758
Female gender	3/10 (30%)	1/10 (10%)	-
NYHA class	2.40 ± 0.38	2.00 ± 0.32	0.453
Atrial fibrillation rhythm	3 (30%)	2 (20%)	-
LV diastolic dimension, mm	57.60 ± 2.22	59.80 ± 2.29	0.521
LV systolic dimension, mm	35.90 ± 2.05	36.20 ± 1.69	0.916
LV ejection fraction, %	61.80 ± 2.70	66.50 ± 2.10	0.210
Left atrial volume index, mL/m^2^	93.78 ± 9.15	90.60 ± 7.28	0.807
MA dimension, mm	39.90 ± 0.87	34.20 ± 1.77 *	0.016
MA dimension/BSA, mm/m^2^	24.04 ± 1.36	19.81 ± 0.55 *	0.019
Severe mitral regurgitation	9 (90%)	10 (100%)	-
Mitral regurgitant volume, mL	92.00 ± 12.36	100.44 ± 11.38	0.649
Effective regurgitant orifice, cm^2^	0.67 ± 0.11	0.71 ± 0.09	0.825
AML length, mm	31.90 ± 1.65	23.44 ± 0.87 *	0.0008
AML thickness, mm	4.68 ± 0.38	2.86 ± 0.11 *	0.0013
PML length, mm	21.10 ± 1.67	18.22 ± 1.40	0.238
PML thickness, mm	4.49 ± 0.38	3.56 ± 0.39	0.133

BSA: Body surface area; LV: left ventricular; MA: mitral annular; AML: anterior mitral leaflet; PML: posterior mitral leaflet. Values presented are mean ± SEM or numbers (percentages), Student’s *t*-test (type 3); *: *p* < 0.05.

**Table 2:** Inversed expression patterns between microRNA and putative target genes. (Proteoglycans).

ID	Fold Change (FED/MMVP)	ID	Fold Change (FED/MMVP)
Decorin (DCN)	0.6	hsa-miR-203	2.1
		hsa-miR-505	2.4
		hsa-miR-532-3p	1.7
		hsa-miR-770-5p	1.2
Aggrecan (ACAN)	0.5	hsa-miR-1273e	1.8
		hsa-miR-203	2.1
		hsa-miR-532-3p	1.7
		hsa-miR-646	2.2
		hsa-miR-770-5p	1.2
		hsa-miR-939	2.3
Fibromodulin (FMOD)	0.4	hsa-miR-1273e	1.8
		hsa-miR-17	1.7
		hsa-miR-203	2.1
		hsa-miR-28	1.5
		hsa-miR-532-3p	1.7
		hsa-miR-770-5p	1.2
		hsa-miR-939	2.3

Putative interactions between microRNA and 3′UTR of genes were predicted by *in silico* microRNA-mRNA prediction database, miRWalk. Data presented are the fold change of genes in FED cohort relative to the average expression levels from MMVP cohort. Only the expression of genes that showed statistical difference between MMVP and FED were listed.

**Table 3:** Inversed expression patterns between microRNA and their target genes. (Structural integrity and cellular specific markers).

ID	Fold Change (FED/MMVP)	ID	Fold Change (FED/MMVP)
ACTA2	0.6	hsa-miR-17	1.7
		hsa-miR-28-5p	1.5
ECM2	0.7	hsa-miR-203	2.1
DES	10.3	hsa-miR-423-5p	0.6
		hsa-miR-543	0.6
ESM1	0.15	hsa-miR-203	2.1
		hsa-miR-28-5p	1.5
		hsa-miR-505	2.4
		hsa-miR-532-3p	1.7
		hsa-miR-646	2.2
VIM	1.4 ^#^	hsa-miR-19b	0.6

Putative interactions between microRNAs and 3′untranslated regions of genes were predicted by the *in silico* microRNA-mRNA prediction database, miRWalk. Data presented are the fold change of gene expressions in FED patients relative to the average expression levels from MMVP patients. The expression of genes that showed statistical difference (*p* < 0.05) between MMVP and FED were listed except VIM, ^#^: Student’s *t*-test (type 3), *p* = 0.055.

## Supplementary Materials

Supplementary materials can be found at http://www.mdpi.com/1422-0067/17/5/753/s1.

## Abbreviations

MMVP	myxomatous mitral valve proplase
FED	fibroelastic deficiency disease
DMVD	degererative mitral valve disease
VHD	valvular heart disease
3′UTR	3′ end untranslated region
DIANA	DNA intelligent Analysis
KEGG pathways	Kyoto Encyclopedia of Genes and Genomes pathways
DCN	decorin
ACAN	aggrecan
FMOD	fibromodulin
LUM	lumican
HSPG2	heparin sulfate proteoglycan 2
VCAN	versican
BGN	biglycan
ACTA2	α actin 2
ECM2	extracellular matrix protein 2
COL1A2	colleagen type 1 α 2
COL2A1	colleagen type 2 α 1
COL6A2	colleagen type 6 α 2
DES	desmin
VIM	vimentin
ESM1	endothelial cell specific molecule 1
PECAM1	platelet/ endothelial cell adhesion molecule 1
VIC	valvular interstitial cell
