# Supplementary material for: Differential MicroRNA Expression Profile in Myxomatous Mitral Valve Prolapse and Fibroelastic Deficiency Valves
Source: Int J Mol Sci. 2016 May 18;17(5):753. doi: 10.3390/ijms17050753 (PMC4881574; doi:10.3390/ijms17050753)
Supplement: Supplementary file 1 [file ijms-17-00753-s001.pdf]

Supplementary Materials: Differential MicroRNA Expression Profile in Myxomatous Mitral Valve Prolapse and Fibroelastic Deficiency Valves

Yei-Tsung Chen, Juan Wang, Abby S.Y. Wee, Quek-Wei Yong, Edgar Lik-Wui Tay, Chin Cheng Woo, Vitaly Sorokin, Arthur Mark Richards and Lieng-Hsi Ling

Table S1. Detailed characterization of individual patients with myxomatous mitral valve prolase (MMVP) and fibroelastic deficiency (FED).

Case	Prolapsed Segment(s)	Flail Segment	Segment with Ruptured Chord	Myxomatous, or Redundant Mitral Leaflets	AML Length, mm	AML Thickness, mm	PML Length, mm	PML Thickness, mm
MMVP1	A2 A3 P1 P2 P3	P2	P2	yes	30	4	24	4.8
MMVP2	A2 A3 P1 P2 P3	none	none	yes	30	4	13	1.8
MMVP3	A1 A2 A3 P3	none	none	yes	46	6	16	6
MMVP4	none	P1 P2	P2	yes	29	5	23	6
MMVP5	A2 A3 P2 P3	none	none	yes	31	5.1	20	4.8
MMVP6	P2	none	none	yes	32	3.3	26	3.6
MMVP7	A2 A3 P1 P2 P3	none	none	yes	33	6.8	22	4
MMVP8	A2 A3	none	none	yes	34	5.5	14	5
MMVP9	P3 A3 A2	P2	P2	yes	28	4.6	31	5.2
MMVP10	P1 P2 P3 A2 A3	none	no	yes	26	2.5	22	3.7
FED1	none	P3	P3	no	28	2.9	16	3.2
FED2	P3	P2	P2	no	22	2.7	17	1.9
FED3	A2	P2	none	no	26	3.1	22	5
FED4	none	P2	P2	no	23	3.1	19	3.1
FED5	P2	P3	P3	no	-	-	-	-
FED6	none	P2	P2	no	19	2.7	20	4
FED7	P3	P2	P2	no	20	2.5	16	4.7
FED8	none	C2	C2	no	26	3.6	17	3.6
FED9	A3 P3	A2	A2	no	24	2.6	10	1.4
FED10	none	P2	P2	no	23	2.5	27	5.1

A (1, 2 or 3), anterior leaflet segment affected, AML, anterior mitral leaflet; P (1, 2 or 3), posterior leaflet scallop; PML, posterior mitral leaflet. For patient FED5, leaflet measurements were omitted as the original echocardiogram was no longer available.

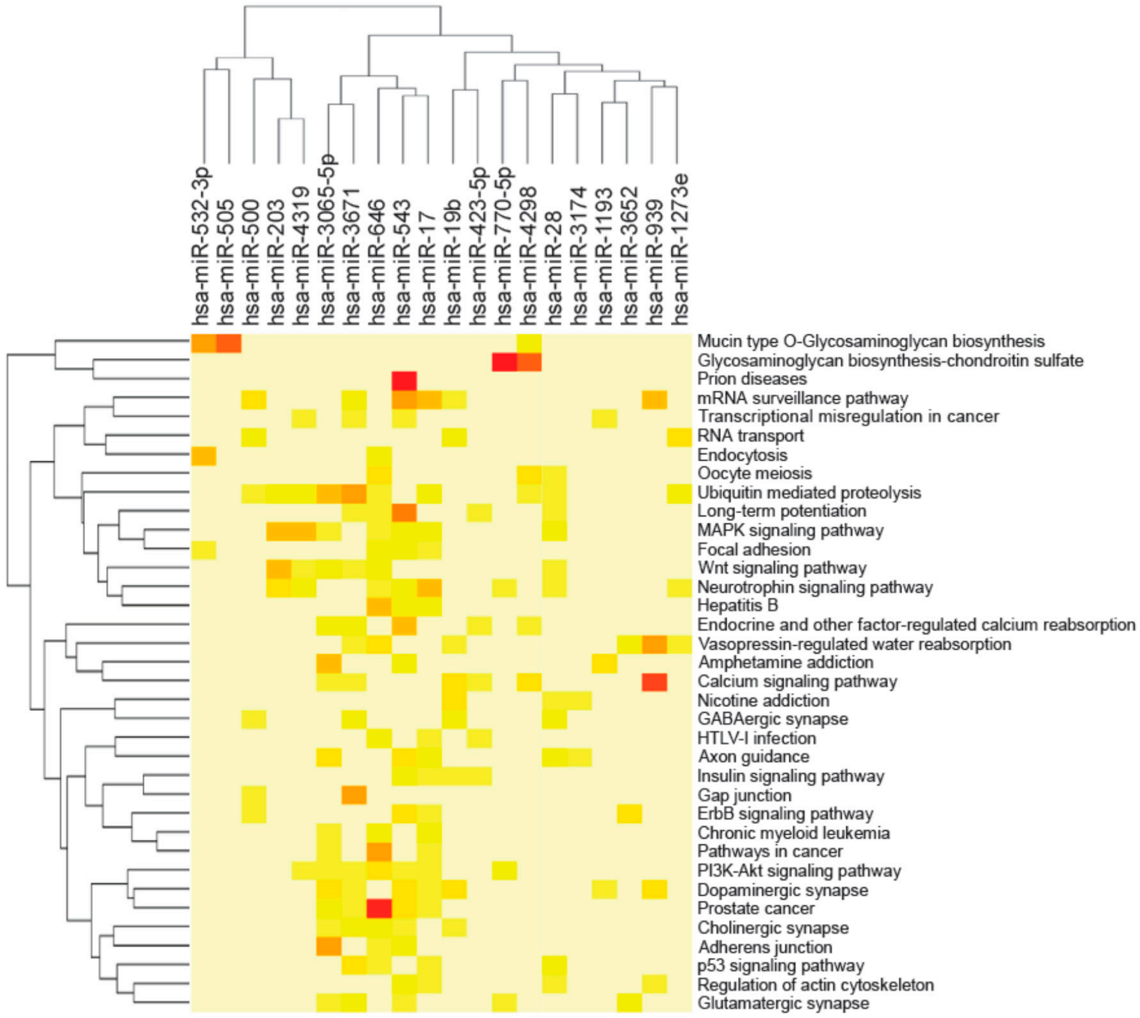

Figure S1. Heat map of microRNAs versus predicted pathways. Hierarchical clustering of predicted pathway and microRNAs that differentially expressed in MMVP and FED cohorts. The darker color clocks represent lower *p* values (cut-off, $p < 0.05$; Fisher's method).

Table S2. The Kyoto Encyclopedia of Genes and Genomes (KEGG) Pathway predication.

KEGG Pathway (Pathway Union)	*p*-Value	#Genes	#miRNAs
Ubiquitin mediated proteolysis	2.15×10^{-12}	59	10
Transcriptional misregulation in cancer	3.27×10^{-12}	53	6
MAPK signaling pathway	4.05×10^{-10}	69	7
Neurotrophin signaling pathway	1.54×10^{-9}	36	8
Dopaminergic synapse	3.15×10^{-8}	46	7
Calcium signaling pathway	8.13×10^{-8}	53	6
Prostate cancer	1.24×10^{-7}	35	5
Wnt signaling pathway	4.08×10^{-7}	41	6
Glycosaminoglycan biosynthesis—chondroitin sulfate	1.60×10^{-6}	2	2
Long-term potentiation	5.18×10^{-6}	25	5
Mucin type O-Glycan biosynthesis	1.08×10^{-5}	5	3
Vasopressin-regulated water reabsorption	4.82×10^{-5}	14	6
PI3K-Akt signaling pathway	0.0001	99	7
Axon guidance	0.0001	37	5
Amphetamine addiction	0.0003	22	3

Table S2. *Cont.*

KEGG Pathway (Pathway Union)	*p*-Value	#Genes	#miRNAs
Glutamatergic synapse	0.0004	34	5
Prion diseases	0.0004	3	1
ErbB signaling pathway	0.0007	24	4
Endocrine and other factor-regulated calcium reabsorption	0.0007	18	5
Regulation of actin cytoskeleton	0.0013	44	4
Focal adhesion	0.0017	43	4
Gap junction	0.0023	16	2
Hepatitis B	0.0024	32	3
Oocyte meiosis	0.0044	17	3
Endocytosis	0.0052	24	2
Insulin signaling pathway	0.0059	27	4
RNA transport	0.0101	15	3
mRNA surveillance pathway	0.0125	25	4
GABAergic synapse	0.0142	18	4
Cholinergic synapse	0.0170	30	5
Nicotine addiction	0.0196	6	3
p53 signaling pathway	0.0229	18	4
Pathways in cancer	0.0251	53	3
HTLV-I infection	0.0328	33	3
Adherens junction	0.0342	25	3
Chronic myeloid leukemia	0.0493	14	3
**KEGG Pathway (Gene Union)**			
Ubiquitin mediated proteolysis	1.48×10^{-23}	65	18
Prostate cancer	1.19×10^{-16}	42	16
Calcium signaling pathway	8.66×10^{-16}	75	18
ErbB signaling pathway	1.96×10^{-15}	41	17
Transcriptional misregulation in cancer	4.71×10^{-14}	75	19
Long-term potentiation	1.32×10^{-13}	33	19
MAPK signaling pathway	3.90×10^{-13}	100	19
Endocrine and other factor-regulated calcium reabsorption	1.28×10^{-12}	23	14
Dopaminergic synapse	1.84×10^{-12}	55	19
Glutamatergic synapse	2.53×10^{-11}	50	18
PI3K-Akt signaling pathway	3.96×10^{-11}	120	19
Endocytosis	4.68×10^{-11}	78	18
Insulin signaling pathway	7.77×10^{-11}	55	18
Gap junction	2.00×10^{-10}	37	16
Regulation of actin cytoskeleton	2.93×10^{-10}	82	19
Neurotrophin signaling pathway	3.76×10^{-10}	50	20
Wnt signaling pathway	9.20×10^{-10}	62	19
Focal adhesion	1.11×10^{-9}	75	19
mTOR signaling pathway	3.46×10^{-9}	29	15
Axon guidance	4.67×10^{-9}	56	19
mRNA surveillance pathway	2.62×10^{-8}	38	17
p53 signaling pathway	2.26×10^{-7}	29	18
Melanogenesis	3.64×10^{-7}	40	17
GnRH signaling pathway	5.03×10^{-7}	36	18
TGF-beta signaling pathway	2.71×10^{-6}	35	14
Fc gamma R-mediated phagocytosis	4.65×10^{-6}	36	18

Table S2. *Cont.*

KEGG Pathway (Gene Union)	*p*-Value	#Genes	#miRNAs
Retrograde endocannabinoid signaling	1.14×10^{-5}	44	17
Type II diabetes mellitus	1.14×10^{-5}	21	13
Prion diseases	1.61×10^{-5}	10	7
Aldosterone-regulated sodium reabsorption	1.84×10^{-5}	17	12
Dilated cardiomyopathy	2.51×10^{-5}	35	14
Endometrial cancer	4.33×10^{-5}	21	12
Cholinergic synapse	4.51×10^{-5}	45	17
Melanoma	8.49×10^{-5}	28	15
Glycosylphosphatidylinositol(GPI)-anchor biosynthesis	0.0001	11	10
Protein processing in endoplasmic reticulum	0.0001	61	19
Chronic myeloid leukemia	0.0001	28	15
RNA transport	0.0002	54	20
B cell receptor signaling pathway	0.0003	28	16
Colorectal cancer	0.0003	25	11
Small cell lung cancer	0.0004	31	14
HTLV-I infection	0.0004	87	20
Long-term depression	0.0005	27	12
Inositol phosphate metabolism	0.0005	25	15
Hypertrophic cardiomyopathy (HCM)	0.0006	31	13
Adherens junction	0.0006	33	15
Taurine and hypotaurine metabolism	0.0008	5	6
Gastric acid secretion	0.0008	28	15
T cell receptor signaling pathway	0.0008	38	16
GABAergic synapse	0.0008	39	17
Pathways in cancer	0.0010	112	19
Nicotine addiction	0.0014	19	13
Hepatitis C	0.0014	45	17
RNA degradation	0.0015	27	17
Glioma	0.0016	29	14
Phosphatidylinositol signaling system	0.0030	34	16
Adipocytokine signaling pathway	0.0033	25	14
Oocyte meiosis	0.0033	45	18
Hepatitis B	0.0061	52	20
Vasopressin-regulated water reabsorption	0.0061	16	12
HIF-1 signaling pathway	0.0067	37	15
Non-small cell lung cancer	0.0079	21	11
Salivary secretion	0.0081	30	17
VEGF signaling pathway	0.0104	23	17
Osteoclast differentiation	0.0161	42	17
Jak-STAT signaling pathway	0.0179	48	19
Basal transcription factors	0.0185	16	9
Amphetamine addiction	0.0185	29	16
Circadian rhythm	0.0199	13	10
Maturity onset diabetes of the young	0.0228	10	6
Pancreatic cancer	0.0305	26	14
Acute myeloid leukemia	0.0311	19	12
Chemokine signaling pathway	0.0337	57	18

The DNA intelligent Analysis (DIANA) miRPath v.2.0, a web-based microRNA-targeted pathway analysis algorithm was used to generate the list.

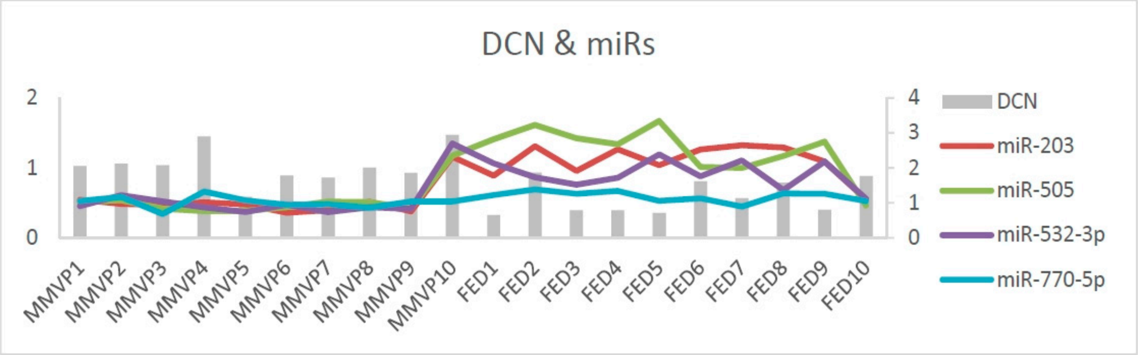

(**a**)

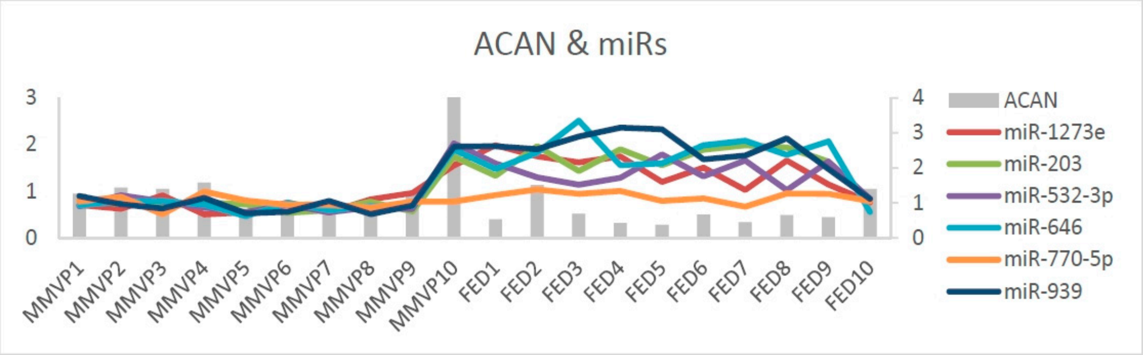

(**b**)

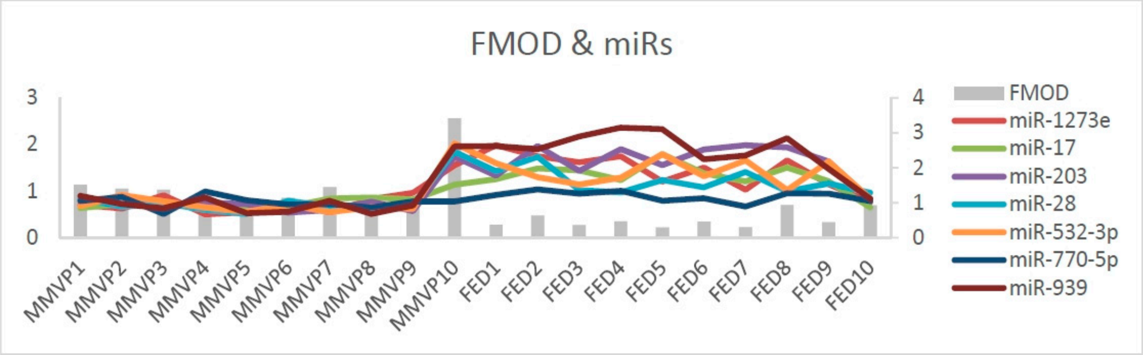

(**c**)

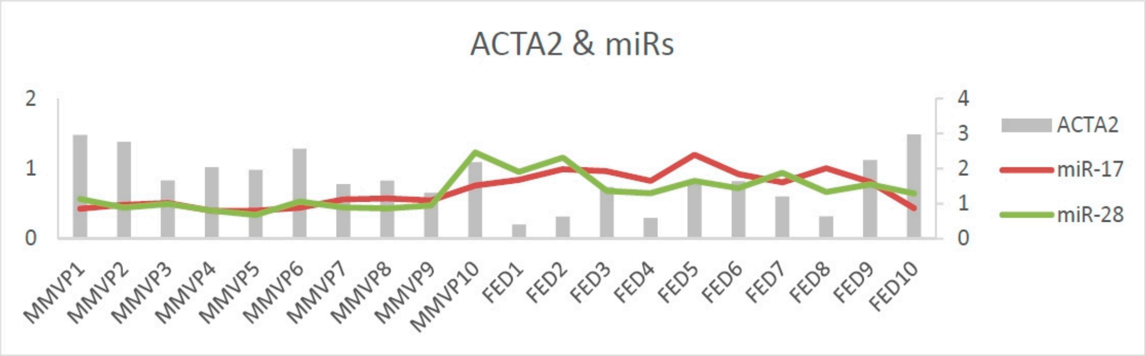

(**d**)

Figure S2. *Cont.*

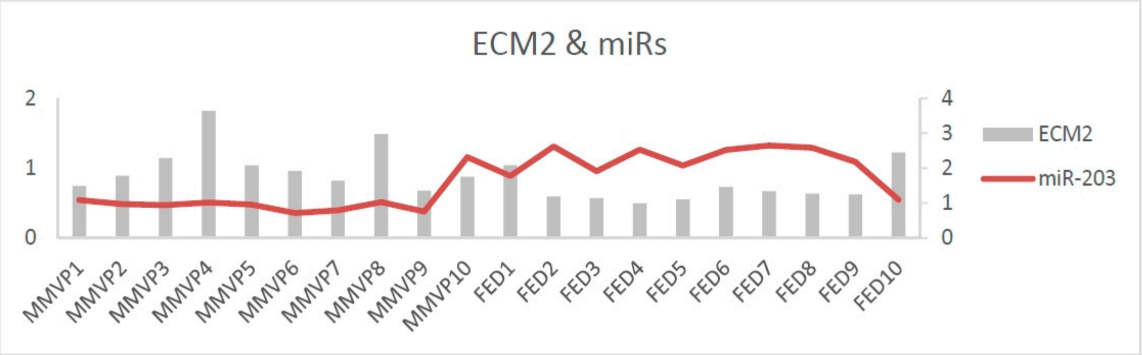

(**e**)

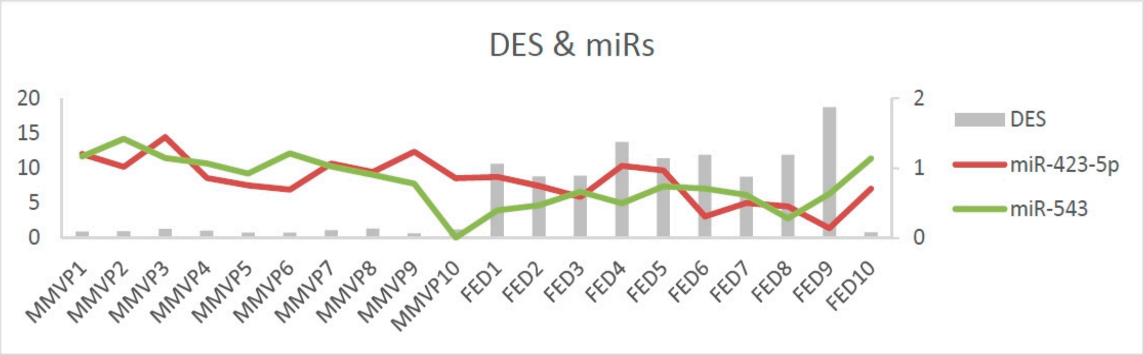

(**f**)

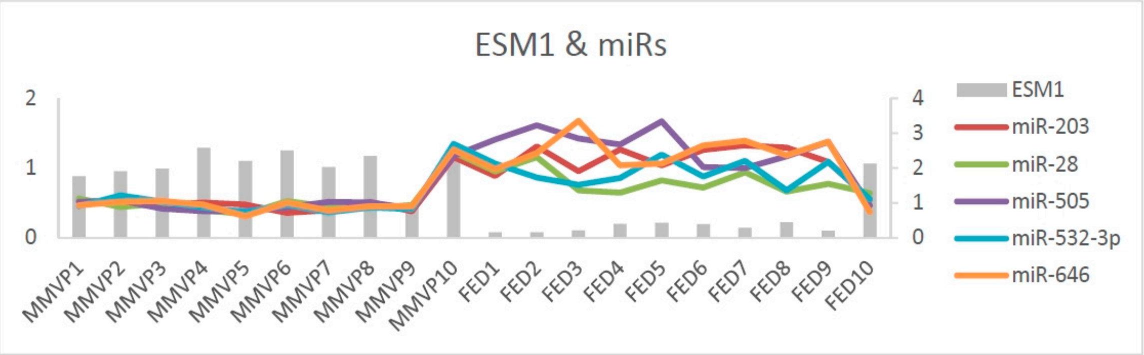

(**g**)

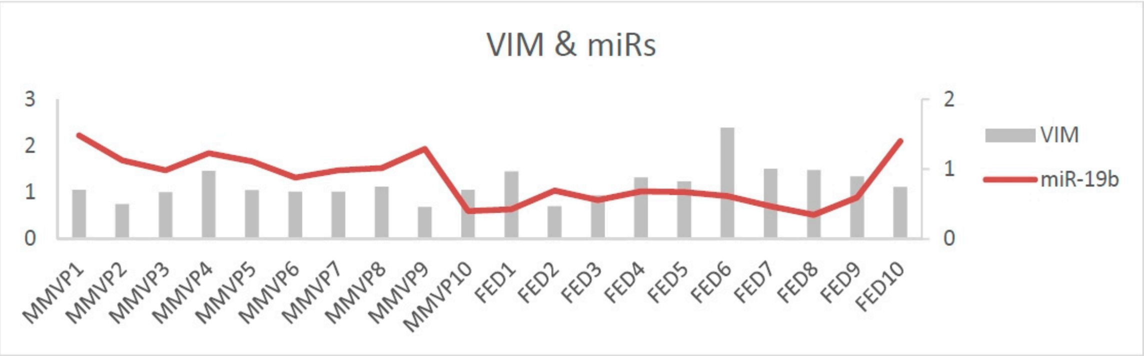

(**h**)

Figure S2. Relative expression patterns between microRNAs and putative target genes of individual patients. Data presented are means of gene expression levels relative to the average values of MMVP samples. Primary axis (**left**) indicate the relative expression levels of the predicated target genes (gray columns); whereas, secondary axis (**right**) indicate the relative expression levels of microRNAs (colored lines). (**a**) DCN; (**b**) ACAN; (**c**) FMOD; (**d**) ACTA2; (**e**) ECM2; (**f**) DES; (**g**) ESM1; (**h**) VIM.
